# Ligand-directed labeling of opioid receptors for covalent attachment of fluorophores or small-molecule probes

**DOI:** 10.1016/j.xpro.2023.102231

**Published:** 2023-04-26

**Authors:** Hayden Adoff, Victoria S. Halls, Emily Holland, Braden Lobingier, Seksiri Arttamangkul

**Affiliations:** 1 Chemical Physiology and Biochemistry, School of Medicine, Oregon Health and Science University, Portland, OR, USA; 2 Medicinal Chemistry Core Facility, Oregon Health and Science University, Portland, OR, USA; 3 Vollum Institute, Oregon Health and Science University, Portland, OR, USA

**Keywords:** Cell Biology, Cell culture, Flow Cytometry/Mass Cytometry, Microscopy, Neuroscience, Molecular/Chemical Probes, Chemistry

## Abstract

This protocol describes endogenous labeling of opioid receptors (ORs) using a ligand-directed reagent, naltrexamine-acylimidazole compounds (NAI-X). NAI acts by guiding and permanently tagging a small-molecule reporter (X)—such as fluorophores or biotin—to ORs. Here we detail syntheses and uses of NAI-X for OR visualization and functional studies. The NAI-X compounds overcome long-standing challenges in mapping and tracking endogenous ORs as the labeling can be done *in situ* with live tissues or cultured cells.

For complete details on the use and execution of this protocol, please refer to Arttamangkul et al.^1^^,^^2^

## Highlights

• Fluorescently labeling of opioid receptors using a chemical approach
• Chemical synthesis of the labeling reagent
• Labeling of opioid receptors in heterologous cell lines
• Visualization of endogenous opioid receptors in rodent brain slices

## Abstract (editor highlights)

Publisher’s note: Undertaking any experimental protocol requires adherence to local institutional guidelines for laboratory safety and ethics.

## Graphical abstract

**Figure:**
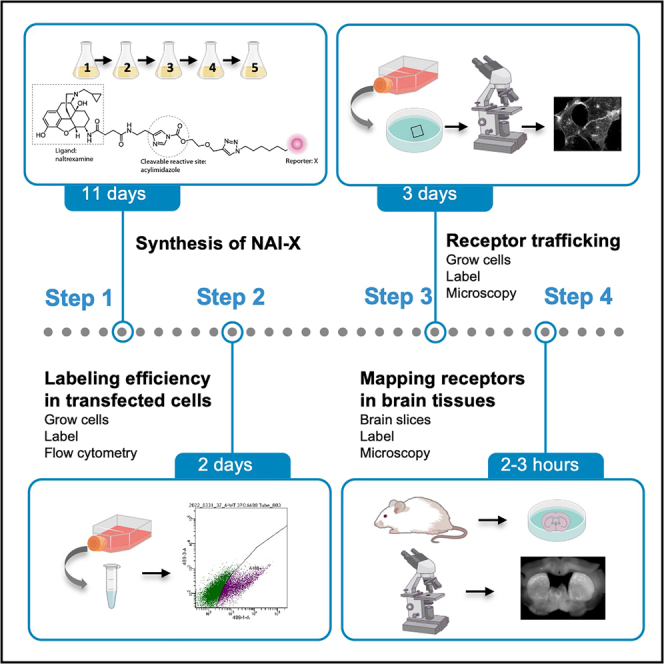

## Before you begin

**Figure 1:**
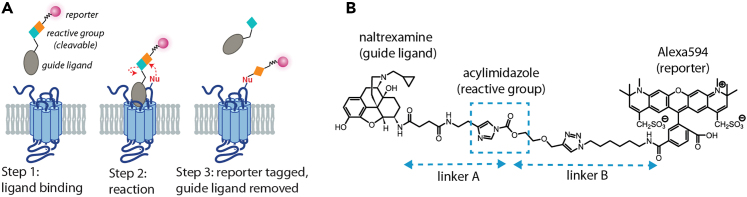
NAI-X compounds (A) Steps of ligand-directed labeling approach.^1^ (B) Outline Naltrexamine-acylimidazole-Alexa594 structure and the role of each component.^2^

Visualization of functional opioid receptors (ORs) in brain tissues from wild type animals has been challenging due to naturally low receptor expression levels. One frequently used approach is to genetically tag ORs with epitope peptides or fluorescent proteins on the N- or C-terminus of the receptors, and that knock-in mice have been made with these approaches.^3^ The development of a chemical labeling approach that resulted in naltrexamine-acylimidazole compounds (NAIs) offers alternative ways to study endogenous ORs from any cells or animals.^1^^,^^2^ The labeling of ORs with NAI compounds relies on a high affinity antagonist naltrexamine to initiate binding at the orthosteric pocket of receptor, which then guide a chemical bond to take place between the reactive acylimidazole moiety of NAI and a nucleophile of amino acid side chains including Lys, Ser, Thr, Tyr or His on the receptor (Figure 1A). The reaction yields a permanent attachment of a reporter or fluorescent dye to the receptor. One significant advantage of this approach is the guide-ligand naltrexamine can be simply washed from the receptor binding pocket with any buffers. The targeted nucleophilic amino acids for labeling reside on the extracellular side of receptor and thus the attachment of a dye or reporter at one of these sites is predicted to have minimal perturbation on receptor activation and regulation processes.

NAI compounds are designed based on ligand-directed affinity approach.^4^^,^^5^ Naltrexamine is a selective OR antagonist and binds equally well to all three opioid receptor subtypes i.e., mu (MOR), delta (DOR) and kappa (KOR). Acylimidazole, which joins naltrexamine and a reporter molecule such as fluorescent dyes or biotin (Figure 1B) has moderated reactivity toward nucleophiles therefore increases specificity towards ORs via high affinity of naltrexamine.^6^ Importantly, the hydroxyl on NAI is also a nucleophile and able to react with acylimidazole and thus potentially cause degradation of the compound. This protocol details the synthesis and specific labeling of NAI compounds for visualization and functional studies of ORs.^1^^,^^2^

### Prepare stable transfection of opioid receptors into cultured cells

**Figure 2:**
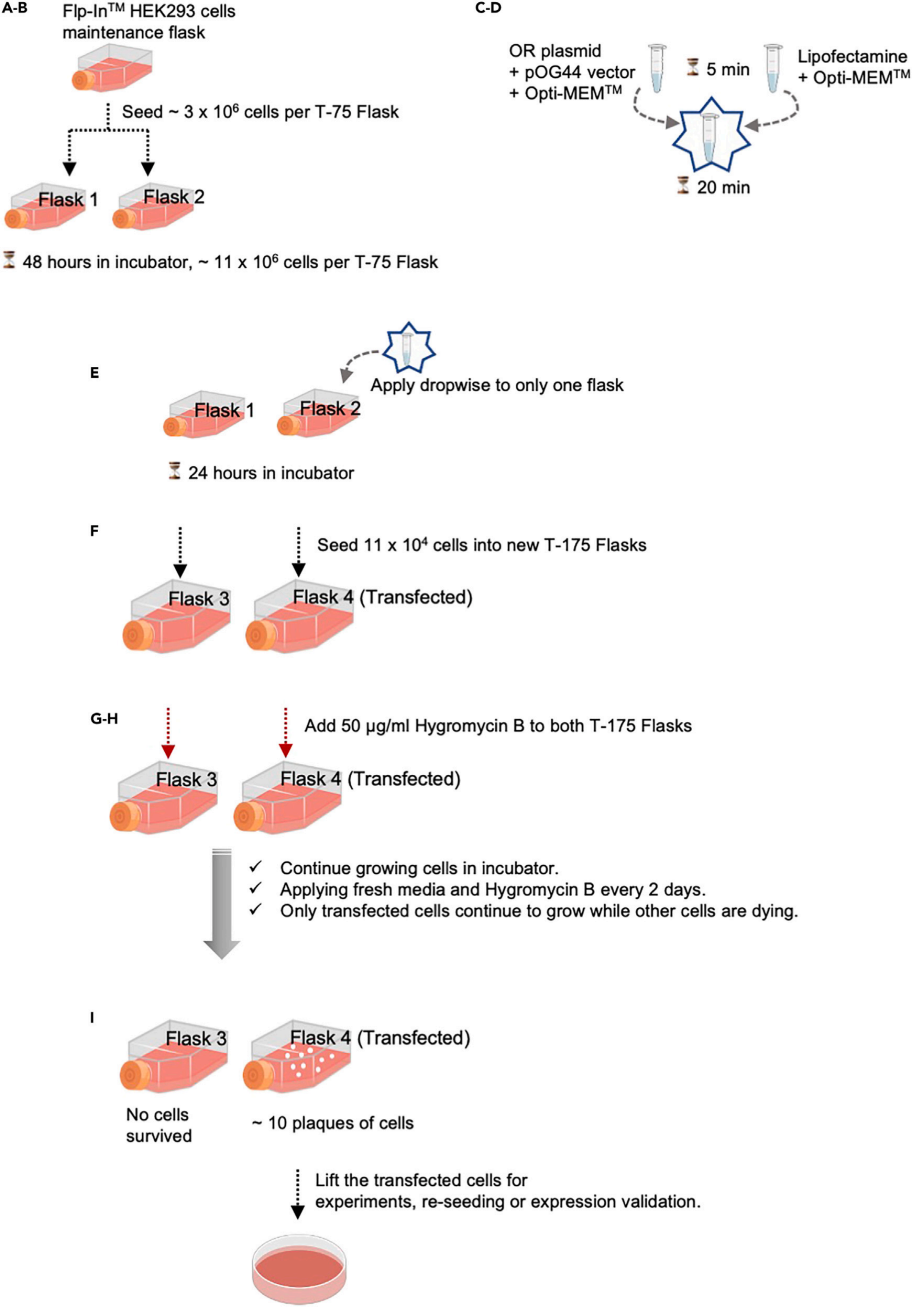
A schematic of stable transfection protocol using HEK293 Flp-In^TM^ cells

The preferred approach for initial characterization of NAI-X compounds is to work with cell lines stably expressing opioid receptors, as this reduces experimental variability by ensuring the same receptor expression levels in all cells. We describe the Invitrogen™ Flp-In™ System here (Figure 2) as it is a straightforward, commercially available, and has several advantages: 1) a single FRT site in the genome allows for the same expression level of the transgene in every clone (isogenic); 2) pooling multiple clones (e.g., polygenic background) avoids the “clone bias” problem observed when the phenotype of a single clone does not reflect the parent cell line, nor the transgene, but a separate gene or genomic feature which became altered in the process of isolating that single clone.

1. Thaw Invitrogen™ HEK293 Flp-In™ cells and culture with Gibco High-Glucose DMEM supplemented with 10% FBS in a 37°C incubator with 5% carbon dioxide.
2. Maintain cells for 1–2 passages (2–4 days).
3. Seed ∼3 × 10^6^ cells (∼10% confluence) in two separate T-75 flasks such that cells will grow in number to about ∼11 × 10^6^ cells (35% confluence) 48 h later.

***Note:*** One flask will remain un-transfected as a means to determine the duration of antibiotic treatment.

4. Once your flask has reached desired number of cells ∼11 × 10^6^ (35% confluence), transfect cells by preparing a transfecting solution using a ∼1:9 ratio of OR-expressing plasmid to FLP-recombinase-expressing vector (pOG44).
  a. In one Eppendorf tube, combine 0.9 μg of the OR-encoding plasmid with Hygromycin B resistance, 7.9 μg pOG44 vector, and 1.75 mL room temperature Opti-MEM™.
  b. In another Eppendorf tube, combine 17.5 μL lipofectamine reagent and another 1.75 mL of room temperature Opti-MEM™.
  c. Incubate each mixture at room temperature for 5 min.
  d. Combine both mixtures and incubate for an additional 20 min at room temperature.
5. Slowly add the transfecting solution dropwise to only one of the T-75 flasks—the one that is designated to be stably transfected.

***Note:*** The other untransfected T-75 flask of cells will be used to determine effectiveness of antibiotic Hygromycin B during selection.

6. 24 h after transfecting, passage cells from each flask to new T-175 flasks with ∼11 × 10^6^ cells (∼15% confluence).

***Note:*** This step has two goals. First, by passaging half of the cells, the genetic diversity from the initial transfection is maintained and potential issues with clone bias are minimized. Second, cells are seeded at a low density so that they do not become confluent during the antibiotic selection.

7. Add 50 μg/mL Hygromycin B to the media, Gibco High-Glucose DMEM plus 10% FBS, to each flask.
8. Replace media including fresh antibiotic every two days while cells are actively dying.

***Note:*** This process typically lasts 4–6 days.

9. Once cells are no longer actively dying, swap media with antibiotic twice per week.
10. Once the un-transfected flask appears to have no remaining cells and the transfected flask has at least 10 plaques, passage cells for further experimentation, re-seeding, and/or expression validation.

***Note:*** Each plaque is a grouping of clonal cells, all touching, that become visible to the unaided eye upon visual inspection of the flask. The process of FLP-mediated recombination is well known to have low efficiency. In our hands, transfection of a T-75 (and selection in a T-175) results in 10–30 plaques if the construct has minimal to no cellular toxicity. Success of transfection can be measured by recombinant tag systems, like the Flag-tag system used in our recombinant OR expression vectors. We generally label cells with anti-flag M1 antibody directly conjugated with AlexaFluor 647 in the presence of calcium and readout fluorescently labelled receptors by flow cytometry.

### Prepare acute brain slices from rodents

11. Anaesthetize a rat or mouse with isoflurane and put down by thoracic percussion or decapitation.

***Note:*** NAI-X labels ORs of any species that bind to naltrexamine. The labeling is expected to be widely useful for any developmental ages or sexes. Examples shown in this protocol use rat or mouse brains from both male and female age 21–28 days.

12. Remove skull and extract the brain.
13. Placed brain in warm (30°C–34°C) oxygenated artificial cerebrospinal fluid (ACSF) buffer solution containing 3 μM (+) MK-801

***Note:*** (+) MK-801 is an NMDA receptor blocker and used to prevent neurotoxicity during preparation.

14. Trim the brain with a razor blade to proper size that contains an area of interest.

***Note:*** We trim the brain such that it will not take too much time during slicing with a vibratome. It is also important that a block of trimmed brain should be large enough to be stably placed on the vibratome plate. The process generally requires trials and practice to achieve healthy brain slices.

15. Use cyanoacrylate glue (e.g., Krazy glue) to fasten the trimmed brain to a vibratome plate.
16. Placed the trimmed brain on vibratome plate in a cutting chamber filled with the oxygenated warm ACSF (30°C–34°C) containing 3 μM (+) MK801.
17. Slice the brain area of interest at 220- or 280-μm thickness using a vibratome (Leica, Nussloch, Germany).

***Note:*** Thickness of brain slices depends on areas of interest as well as experimental goals. For imaging and electrophysiological purpose, a range of 200–300 μm thickness are suitable for handling and maintain healthiness of brain slices.

18. Allow slices to recover in a vial containing warm (34°C), oxygenated ACSF plus 10 μM (+) MK-801 for 30 min.
19. Transfer slices to oxygenated ACSF without (+) MK-801 until ready to use.

***Note:*** The temperature of ACSF during this step can be varied depending on the area of brain. For example, the temperature at 31°C–34°C is suitable to maintain locus coeruleus and midbrain dopamine slices for 6 h whereas the room temperature (∼22°C) is good for striatal slices.

### Institutional permissions

The use of animals was conducted in accordance with the National Institutes of Health guidelines and the approval of the Institutional Animal Care and Use Committee of OHSU. Animals were separated by sexes and housed as a group (2–3 rats or 2–5 mice) to a cage in the animal care facility. Food and water were available *ad libitum* on the 12-h light/dark cycle*.*

## Key resources table

**Table:** 

REAGENT or RESOURCE	SOURCE	IDENTIFIER
**Antibodies**		

Monoclonal ANTI-FLAG® M1 antibody, mouse	MilliporeSigma	SKU# F3040-.2MG

**Chemicals, peptides, and recombinant proteins**		

2-(Prop-2-yn-1-yloxy)ethanol	AstaTech	Cat# F16468
Acetonitrile HPLC grade	Fisher Scientific	Cat# A998-4
Acetonitrile LCMS grade	Fisher Scientific	Cat# A955-4
AFdye 594 azide	Click Chemistry Tools	Cat# 1295-1
AFdye 488 azide	Click Chemistry Tools	Cat# 1275-1
β-Naltrexamine·2HCl	Drug Supply Program (DSP) of National Institute on Drug Abuse	Cat# 9333-010
Deuterated chloroform	Fisher Scientific	Cat# AC209561000
Deuterated methanol	Oakwood Chemical	Cat# 403756
Deuterium oxide	Fisher Scientific	Cat# AC166301000
Dichloromethane	Fisher Scientific	Cat# D3720
Dimethylformamide	Fisher Scientific	Cat# D119-4
Dimethylsulfoxide	Fisher Scientific	Cat# BP231-1
Ethanol	Fisher Scientific	Cat# 04-355-450
Ethyl acetate	Fisher Scientific	Cat# E145-20
Formic acid	Fisher Scientific	Cat# A117-50
HBTU	TCI America	Cat# B1657
Hexane	Fisher Scientific	Cat# H292-20
Histamine·2HCl	Fisher Scientific	Cat# 50-518-980
Methanol	Fisher Scientific	Cat# A41220
*N,N′*-disuccinimidyl carbonate	Fisher Scientific	Cat# D16625G
NAI-A594	This paper and Arttamangkul et al.^1^	
NAI-A488	Arttamangkul et al.^1^	
Naloxone hydrochloride	Tocris	Cat# 0599
(+) MK-801	Hello Bio	Cat# HB0004
Phosphomolybdic acid hydrate	Fisher Scientific	Cat# P191025G
Pyridine	Fisher Scientific	Cat# AC339421000
Succinic anhydride	Fisher Scientific	Cat# S010725G
Tetrakis(acetonitrile)copper(I) tetrafluoroborate	Fisher Scientific	Cat# T26661G
Triethylamine	Fisher Scientific	Cat# MTX120011
Tris(benzyltriazolylmethyl)amine	Fisher Scientific	Cat# T29931G
Water LCMS grade	Fisher Scientific	Cat# W6-4
Poly L-lysine	Sigma-Aldrich	CAT# P8920-100ML
Gibco™ DMEM, high glucose	Gibco	Cat# 11-965-118
Tryple Express Enzyme (1×) no phenol red	Fisher Scientific	Cat# 12-604-021
Fetal bovine serum (FBS)-characterized, 500 mL	Cytiva	Cat# SH 30396.03
Dulbecco’s Phosphate-Buffered Salt Solution 1×	Corning	Cat# 21030CV
Opti-MEM™ I Reduced Serum Medium	Gibco™	Cat# 31985088
Lipofectamine 2000	Invitrogen	Cat# 11668019
Hygromycin B	Gibco™	Cat# 10687010

**Experimental models: Cell lines**		

Flp-In™-293 Cell Line	Invitrogen	Cat# R75007

**Experimental models: Organisms/strains**		

Rat/ Sprague Dawley	Charles River Laboratories	Stock # 001 RRID: RGD_734476
Mouse/ C57BL/6J	Jackson Laboratories	Stock # 000664 RRID: IMSR_JAX:000664
Mouse/C57BL/6j(ChAT-BAC-eGFP)	Jackson Laboratories	Stock#007902 RRID:IMSR_JAX:007902

**Recombinant DNA**		

pUDNA5_SSFDORwt	Novy et al.^7^	
pUDNA5_SSFMORwt	Novy et al.^7^	
pCDNA3.1_SSFDORwt	Lobingier et al.^8^	
pCDNA3.1_SSFMORwt	Polacco et al.^9^	
pOG44	Invitrogen	Cat# V600520

**Software and algorithms**		

FlowJo™ v10.8.1	BD Life Sciences	
Fiji (ImageJ)	On GitHub	License GPLv3+
QCapture v2.98.2	QImaging	

**Other**		

Falcon TM Tissue Culture Treated Flasks, 175 cm^2^	Corning	Cat# 353112
Falcon TM Tissue Culture Treated Flasks, 75 cm^2^	Corning	Cat# 353110
Vibratome	Leica Biosystems	Leica VT1200S

## Materials and equipment

### NAI-X stock solution

To prepare a stock solution:

• Dissolve lyophilized powder of 20 nmol NAI-X in dimethylsulfoxide (DMSO) 2 μL.
• Add 198 μL deionized water.

***Note:*** NAI-X is generally aliquoted and stored as 20 nmol lyophilized powder in an Eppendorf tube. The combined solvent used to make a solution, 200 μL will result a final concentration of 100 μM.

• Make 10 or 20 μL aliquots.

***Note:*** This 100 μM stock solution can be stored at −20°C for up to 6 months. The working solution should be stored at 4°C and used within ∼2 weeks.

**CRITICAL:** The concentration of stock solution of NAI-X should not be higher than 100 μM. At higher concentrations e.g., 200 or 250 μM, NAI-X can degrade and lose activity.

### Artificial cerebrospinal fluid (ACSF) solution

To prepare a liter of ACSF, dissolve the following salts in Milli-Q water. The preparation time of the ACSF is approximately 30 min.

**Table:** 

Reagent	Mol weight	Final concentration (mM)	Amount
NaCl	58.44	136	7.95 g
KCl	74.55	2.5	0.186 g
MgCl_2_, anhydrous	96.21	1.2	0.115 g
CaCl_2_·2H_2_O	147.04	2.4	0.352 g
NaH_2_PO_4_	119.98	1.2	0.144 g
NaHCO_3_	84.01	21.4	1.8 g
Dextrose	180.16	11.1	2.0 g
ddH_2_O	18	N/A	to make 1 L
**Total**	**N/A**	**N/A**	**1 L**

Bubbling the solution with carbogen gas mixture (medical grade) of 5% carbon dioxide and 95% oxygen in a water bath at 34°C for at least 15 min before use.

***Note:*** ACSF should be made fresh daily.

### Macro zoom fluorescence microscope system

We use an Olympus MVX10 macro zoom fluorescence microscope equipped with a Retiga-2000R camera from QImaging. The magnification range is 0.63× – 6.3× with MVPLAPO 1×, N.A. 0.25, air objective lens and WHN10× eyepiece. We use Q-Capture software (QCapture 2.98.2) for Image acquisition.

### Two-photon microscope

The two-photon microscope is a custom-built apparatus using an upright microscope Olympus BX51W1 and a 60× water immersion Olympus LUMFI, NA 1.1 lens. Excitation of laser generated from Cameleon Ti:sapphire is set at 810-nm for Alexa594 dye. We use Scan Image Software^10^ for data acquisition.

### Confocal microscope

We use a Nikon Spinning Disk confocal microscope (Laser: 488 nm, Emission filter: 525/36 nm) equipped with a temperature control system and a 100× oil immersion objective (NA = 1.48, working distance = 0.12 mm).

### Image analysis

We use Fiji software^11^ for all post hoc analysis of images. All images are set using the same brightness/contrast for clarity.

### Flow cytometry

We use a Beckman Coulter Cytoflex S (B78557) to measure fluorescence of labeled cells and FlowJo™ v10.8.1 Software (BD Life Sciences, https://www.flowjo.com/solutions/flowjo) software for data analysis.

## Step-by-step method details

### Synthesis of NAI compounds (NAI-X)

**Timing: 11 days (for steps 1 to 5)**

**Timing: 2 days (for step 1)**

**Timing: 3 days (for step 2)**

**Timing: 1 day (for step 3)**

**Timing: 3 days (for step 4)**

**Timing: 2 days (for step 5)**

There are 5 steps in making NAI compounds.

1. Synthesis of compound 1: 3-{[2-(1H-imidazol-4-yl)ethyl]carbamoyl}propanoic acid.
  Compound 1 (see Reaction equation 1) generated in this step will be used to make acylimidazole component. It also serves as a linker between the guide ligand β-naltrexamine and acylimidazole moiety.
  a. Dissolve succinic anhydride (1.76 g, 17.6 mmol) in 15 mL of anhydrous dimethylformamide (DMF), under an argon atmosphere.
  b. Slowly add a solution of histamine·2HCl (2.93 g, 15.9 mmol) in 25 mL anhydrous DMF and triethylamine (NEt_3_, 43 mL, 31.8 mmol) dropwise.
  c. Stir the solution overnight (12–18 h) at room temperature (22°C–25°C).
  d. Pour the reaction mixture, which becomes a cloudy form, through a fritted glass funnel placed on a filtering flask and apply a house vacuum to filter.
  e. Discard precipitate.
  f. Collect the filtrate into a round bottom flask.
  g. Remove DMF under reduced pressure by applying a vacuum using a Fisher Scientific Maxima C Plus high vac pump connected to a condensing device for liquid collection.
  h. Dissolve the residue in ethanol (20 mL, 200 proof).
  i. Chill the mixture to 4°C overnight in a cold room or fridge.
    ***Note:*** At this point, a cloudy solution is formed. The white solid precipitate is Compound 1 (see Reaction equation 1).
  j. Collect the white solid precipitate by filtering the cloudy solution using a fritted glass funnel placed on a filtering flask and apply a house vacuum to filter.
  k. Wash the solid product 3 times with ice-cold ethanol by adding the solvent to the precipitate in the fritted glass funnel and apply a house vacuum to remove ethanol.
  l. Place the wet solid product in a 20 mL scintillation vial and dry it by applying a house or high vacuum at room temperature.
    ***Note:*** The yield of compound 1 is approximately 84% of starting material (histamine·2HCl).
  m. Take 10 mg of this white powder and dissolve it in deuterium water for NMR analysis: 1H NMR (D_2_O): δ (ppm) 8.5 (s, 1H), 7.20 (s, 1H), 3.41 (t, J = 6.37 Hz, 2H), 2.86 (t, J = 6.37 Hz, 2H), 2.35 (s, 4H).
    ***Note:*** Compound 1 as shown in [Reaction equation 1] can be stored at room temperature for 12 months.

2. Synthesis of compound 2: N-[(1S,5R,13R,14R,17S)-4-(cyclopropylmethyl)-10,17-dihydroxy-12-0xa-4-azapentacyclo[9.6.1.0^1,13^.0^5,17^.0^7,18^]octadeca-7,9,11(18)-trien-14-yl]-N’-[2-(1H-imidazol-4-yl)ethylbutanediamide.
  Compound 2, naltrexamine histamine (see Reaction equation 2) made in this step will be used as an intermediate for generating NAI compounds.
  a. Dissolve compound 1 (20.3 mg, 96 μmol), HBTU (*N,N,N′,N′*-tetramethyl-O-(1H-benzotriazol-1-yl)uranium hexafluorophosphate (27.3 mg, 72 μmol) and triethylamine (2 drop, ∼ 40 μL) in 1 mL DMF.
  b. Stir for 30 min at room temperature (22°C–25°C).
  c. Dissolve β-naltrexamine·2HCl (9.97 mg, 24 μmol) in 0.5 mL DMF.
  d. Transfer the naltrexamine solution to a 2.5 mL microwave vial.
  e. Add solution from step a.
  f. Add another 1 mL DMF to wash down the reagents on the side of vial.
  g. Set a Biotage Initiator^+^ Microwave Reactor to 150°C, 10 min reaction time at normal absorption.
  h. Check the completion of the reaction by HPLC analysis of the crude material using a reverse phase Vydac 300Å C8, 4.6 × 250 mm column, 1 mL/min flow and a linear gradient over 15 min from 0%–40% acetonitrile in water with 0.1% formic acid.
    ***Note:*** To confirm the reaction is completed, the peak of naltrexamine will be absent on the chromatogram.
  i. Pour the reaction mixture into a round bottom flask.
  j. Remove DMF under reduced pressure by applying a vacuum using a Fisher Scientific Maxima C Plus high vac pump connected to a condensing device for liquid collection.
  k. Dissolve the residue, which appears as a film around the round bottom flask with 5% acetonitrile in water plus 0.1% formic acid for HPLC purification.
  l. Purify the crude solution with an Agilent 1260 Infinity II semi-preparative HPLC system using a reverse phase Vydac 300Å C8, 10 × 250 mm column, 5 mL/min flow, and a linear gradient over 25 min from 0%–40% acetonitrile in water with 0.1% formic acid.
  m. Combine pure fractions and lyophilize.
    ***Note:*** The lyophilized, white powder is obtained at approximately 94% yield of starting material (β-naltrexamine·2HCl) and purity of 97% determined by HPLC.
    **CRITICAL:** The high purity of compound 2 as shown in [Reaction equation 2] is important for the reaction in step iv and v. We routinely check the purity of the lyophilized powder with an Agilent 1260 Infinity II analytical HPLC equipped with a Thermo Scientific Velos LTQ mass spectrometer. Using reverse phase Agilent Pursuit XRs 100Å C18, 4.6 × 250 mm column, 1 mL/min flow and a linear gradient over 15 min from 0%–40% acetonitrile in water with 0.1% formic acid, the product peak will appear at 536.5 (m/z+1) on the electron-spray ionized mass spectrum.
  n. Take 10 mg of this white powder and dissolve in deuterated methanol for NMR analysis: 1HNMR (400 MHz, CD_3_OD): δ (ppm) 8.76 (s, 1H), 7.36 (s, 1H), 7.05 (d, J = 8.4 Hz, 1H), 6.90 (d, J = 8.8 Hz, 1H), 4.68 (d, J = 8Hz, 1H), 4.37 (m, 2H), 4.22 (s, 2H), 3.98 (m, 1H), 3.80 (t, J = 4 Hz, 2H), 3.61 (m, 1H), 3.14 (m, 1H), 2.92 (m, 3H), 2.66 (m, 3H), 2.55 (m, 2H), 2.44 (m, 2 H), 1.90 (m, 1H), 1.67 (m, 3H), 1.12 (m, 1H), 0.85 (m, 1H), 0.76 (m, 1H), 0.53 (m, 2H). MS (ESI pos) m/z found 536.5 (M + H).
    ***Note:*** Compound 2 as shown in [Reaction equation 2] can be stored in a desiccator at room temperature for 12 months. The material used for NMR analyses can be recovered by removal of deuterated methanol under reduced pressure.

3. Synthesis of compound 3: 2,5-dioxopyrrolidin-1-yl 2-(pro-2-yn-1-yloxy)ethyl carbonate.
  This step is to generate an active reagent to produce naltrexamine acylimidazole. Compound 3 (see Reaction equation 3) also acts as a linker with alkyne functional group for click reaction.
  a. Dissolve 2-(Prop-2-yn-1-yloxy)ethanol (25.0 mg, 0.25 mmol) and triethylamine (140 μL, 1 mmol) in 3 mL acetonitrile.
  b. Heat to 40°C while stirring, using a magnetic bar and a stirrer/hotplate.
  c. Add *N,N′*-disuccinimidyl carbonate (96 mg, 0.375 mmol) and continue stirring for 2 h at 40°C.
  d. Check the completion of the reaction by thin-layer chromatography (TLC) using Merck TLC Silica gel 60 F_254_ Aluminum sheets, 75% ethyl acetate in Hexane as the mobile phase.
    ***Note:*** We use phosphomolybdic acid stain for visualization. The product spot normally migrates with an approximate retention factor (R_f_) of 0.63.
  e. Remove acetonitrile under reduced pressure on a Heidolph rotary evaporator.
  f. Dissolve the light-amber crude oil in dichloromethane (DCM) for purification.
  g. Purify with silica gel chromatography on a Biotage Isolera One using a Biotage ZIP KP-Sil 10 g column and a stepwise gradient of 83%–94% to 96% ethyl acetate/hexane.
  h. Check fractions for product mass by Flow Injection Mass Spectrometry on a Thermo Scientific Velos LTQ.
    ***Note:*** The product peak will appear at 242.2 (m/z+1) on the electron-spray ionized mass spectrum.
  i. Combine eluent fractions containing the product.
  j. Remove the solvent under reduced pressure using a Heidolph rotary evaporator.
    ***Note:*** The product is clear light-yellow oil and obtained at approximately 97% yield of starting material (2-(Prop-2-yn-1-yloxy)ethanol).
  k. Take 10 mg of this oil and dissolve in deuterated chloroform for NMR analysis: 1HNMR (400 MHz, CDCl_3_): δ (ppm) 4.56 (m, 2H), 4.19 (d, J = 2.38 Hz, 2H), 3.80 (m, 2H), 2.81 (s, 4H), 2.52 (t, J = 2.38 Hz, 1 H).
    ***Note:*** The compound 3 as shown in [Reaction equation 3] can be stored at −20°C for 3 months. The material used for NMR analyses can be retrieved by removal of deuterated chloroform under reduced pressure using a Heidolph rotary evaporator.

4. Synthesis of compound 4: 2-(prop-2-yn-1-yloxy)ethyl 4-[2-(3-{[1s,5R,13R,14R)-4-(cyclopropylmethyl)-10,17-dihydroxy-12-oxa-4-azapentacyclo[9.6.1.0^1,13^.0^5,17^.0^7,18^] octadeca-7,9,11(18)-trien-14-yl]carbamoyl}propanamido)ethyl]-1H-imidazol-1-carboxylate.
  The product of this step is naltrexamine acylimidazole alkyne (NAI-AK, compound 4 in Reaction equation 4). This is the key intermediate compound to generate NAI-X via click reaction with any azide reporters.
  a. Dissolve compound 3 (7 mg, 29 μmol) in 1.7 mL anhydrous DMF.
  b. Add compound 2 (13 mg, 24 μmol) and 3 μL anhydrous pyridine.
  c. Stir the reaction mixture overnight at room temperature under an argon atmosphere.
  d. Remove the solvent under reduced pressure by applying a vacuum using a Fisher Scientific Maxima C Plus high vac pump connected to a condensing device for liquid collection.
  e. Dissolve residue in 10% ACN in water with 0.1% formic acid (FA) for HPLC purification.
  f. Purify the crude with an Agilent semi-preparative HPLC system using a reverse phase Agilent Pursuit XRs 100Å C18, 10 × 250 mm column, 5 mL/min flow and a linear gradient over 25 min from 5%–60% acetonitrile in water with 0.1% formic acid.
  g. Combine pure fractions and lyophilize.
  h. Check the purity of the lyophilized powder with an Agilent 1260 Infinity II analytical HPLC connected to a Thermo Scientific Velos LIQ mass spectrometer using a reverse phase Agilent Pursuit XRs 100Å C18, 4.6 × 250 mm column, 1 mL/min flow and a linear gradient over 15 min from 5%–60% acetonitrile in water with 0.1% formic acid.
    ***Note:*** A product peak will appear at 662.1 (m/z+1) on the electron-spray ionized mass spectrum. Detection can be simultaneously monitored by UV absorption at 218 nm. The retention time of the product is 9.7 min. The lyophilized, white powder is obtained at approximately 25% yield of starting material (compound 2) with 97% purity determined by HPLC.
  i. Take ∼1 mg of this solid powder and dissolve in deuterium methanol for NMR analysis: 1HNMR (400 MHz, CD3OD): δ (ppm) 8.22 (s, 1H), 7.40 (s, 1H), 6.74 (d, J = 2.12 Hz, 1H), 4.57 (m, 3H), 4.24 (d, J = 2.4 Hz, 2H), 3.90 (m, 3H), 3.63 (m, 1H), 3.46 (m, 2H), 3.16 (m, 2H), 2.87 (m, 3H), 2.76 (t, J = 6.83 Hz), 2.70 (m, 2H), 2.49 (m, 4H), 1.87 (m, 1H), 1.66 (m, 4 H), 1.34 (m, 1 H), 1.22 (m, 1H), 0.84 (m, 1H), 0.76 (m, 1H). MS (ESI pos) m/z found 662.1 (M + H).
    **CRITICAL:** Compound 4 as shown in [Reaction equation 4] should be stored in a moisture-controlled container at −80°C because it is very sensitive to moisture and will degrade as the result of self-reaction by phenol-OH on naltrexamine moiety. We recommend using compound 4 promptly in the next step.

5. Conjugation of NAI-AK with Alexa594 azide using copper (I)-catalyzed azide-alkyne cycloaddition (CuAAA) click chemistry.
  This step is designed to bring in a reporter to the guided ligand naltrexamine. The alkyne functional group in NAI-AK (compound 4) reacts with any azide compounds via copper-catalyzed alkyne azide cycloaddition.^12^^,^^13^ It is a simple, selective, and high yield reaction that can take place in various solvents e.g., dimethylsulfoxide, dimethylformalmide, acetonitrile or water. These exceptional properties of click reaction allow the conjugation of NAI-AK to a reporter with minimal byproducts. Alexa594 azide (AFdye 594 azide from Click Chemistry Tools) was used here as an example to generate NAI-A594 (see Reaction equation 5).
  a. Dissolve Alexa594 azide (1 mg, 1.16 μmol) in 450 mL anhydrous dimethylsulfoxide (DMSO).
  b. Add tris(benzyltriazolylmethyl)amine (TBTA, 19 mg, 36 μmol) and tetrakis(acetonitrile)copper(I) tetrafluoroborate (Cu[MeCN]_4_BF_4_, 8 mg, 27 μmol).
    **CRITICAL:** Since the initial publication,^1^ we notice the intermediate naltrexamine-acylimdazole-alkyne (NAI-AK, compound 4) is very unstable in aqueous solution. The click reaction to conjugate fluorescent dyes is therefore done in anhydrous DMSO and tetrakis(acetonitrile)copper(I) tetrafluoroborate is used as a catalyst instead of CuSO_4_ and ascorbate.
  c. Dissolve compound 4 (2.5 mg, 3.78 μmol) in 450 mL anhydrous DMSO and add to the mixture from b.
  d. Stir overnight (12–18 h) under argon.
  e. Check the completion of the reaction by HPLC analysis of the crude material with an Agilent 1260 Infinity II analytical HPLC connected to a Thermo Scientific Velos LTQ mass spectrometer. The analytical HPLC condition uses a reverse phase Agilent Pursuit XRs 100Å C18, 4.6 × 250 mm column and 1 mL/min flow of a linear gradient over 15 min from 5%–100% acetonitrile in water with 0.1% formic acid.
    ***Note:*** The reaction is completed when Alexa594 azide peak is absent on the chromatogram and the product peak appears at the retention time of 10.6 min.
  f. Purify the reaction mixture on an Agilent Infinity preparative HPLC using a reverse phase Agilent Pursuit XRs 100Å C18, 30 × 250 mm column, 45 mL/min flow and a linear gradient over 15 min from 5%–65% acetonitrile in water with 0.1% formic acid.
    ***Note:*** Any preparative columns can be used for purification with appropriate adjustment of the flow of the solvents. We recommend using a similar packing material to obtain the best separation.
  g. Combine pure fractions and lyophilize overnight (12–18 h).
    ***Note:*** The lyophilized, purple powder is obtained at approximately 98% yield with 75% purity determined by HPLC.
  h. Check the purity of the lyophilized powder with an Agilent 1260 Infinity II analytical HPLC connected to a Thermo Scientific Velos LTQ mass spectrometer using a reverse phase Agilent Pursuit XRs 100Å C18, 4.6 × 250 mm column, 1 mL/min flow and a linear gradient over 15 min from 5%–60% acetonitrile in water with 0.1% formic acid. A product peak will appear at 755.46 (m/z+2) on the electron-spray ionized mass spectrum.
    ***Note:*** UV absorption can also be used to monitor the product at 220 and 594 nm. Dual wavelength detection is recommended.
    **CRITICAL:** If only UV detection is available, it is important to check the combined product fractions with mass spectrometry.
  i. Dissolve the lyophilized product in ∼2 mL acetonitrile/water (1:1) with 0.1% formic acid.
    **CRITICAL:** When using other dyes, it may be advantageous to use acetonitrile/water (1:1) with 0.1% trifluoroacetic acid to ensure better solubility after lyophilization.
  j. Dilute 10 μL with 990 μL methanol.
  k. Measure the absorbance at 590 nm.
  l. Calculate concentration using c = (A/(εL))∗f.
    ***Note:*** c = concentration (Molarity), A = Absorbance, ε = extinction coefficient (88000 M^-1^ cm^-1^ for Alexa594), L = light path length (cm), f = dilution factor (100 as shown in protocol step j).
    **CRITICAL:** If absorbance is above 1, make a new dilution until absorbance is below or in the linear range of Beer’s Law. This value is important for concentration calculations, which is used to determine the total yield and the amount of material (in μL) when aliquoting into smaller portions for storage.
  m. Aliquot in 20 nmol portions and lyophilize.
    ***Note:*** The scale of this synthesis is based on the commercially availability of 1 mg of the dye. Generally, < 1 mg of product is obtained. Weighing out for making a working stock solution is not practical. The best way to get the proper amount of product for easy use and storage is thus relying on their maximal absorption and the known extinction coefficient. We find that 20 nmol material is sufficient for most applications and that the stock and working solution will be consumed within a practicable time.
    **CRITICAL:** The small aliquot of 20 nmol dried compound should be stored at −20°C with humidity control. See the reconstitution procedure and how to store the working solution in Materials and Methods above.

### Validation of NAI-X labeling of opioid receptors in cultured cells

**Timing: 2 days (for step 6)**

**Timing: ∼3 days (For step 7)**

Optimization of the concentration and time used for NAI-X labeling can be guided by labeled cells’ fluorescent signal to background ratio (i.e., labeled in the presence of a blocking ligand such as naloxone). For many experimental applications, we have found that a sub-saturating concentration close to EC_50_ of NAI-X is adequate to efficiently label the receptor. However, a new assay scheme (i.e., new conjugated fluorophore or cell type) may require re-optimization of NAI-X labeling conditions. Ultimately, decision making for NAI-X labeling protocols can be influenced by OR expression levels, fluorescent dyes, and sensitivity of the detection methods required by the experimental question. For general purposes, NAI-X concentrations can range from 10 nM to 500 nM with incubation timing of 1–4 h depending on concentration of the compound and temperature. Therefore, a concentration response curve is performed to identify optimal labeling conditions. This protocol uses HEK293 cells stably expressing the μ-opioid receptors (MOR) as an example.

6. Using flow cytometry to determine specific and irreversible labeling.
  a. Seed 3 × 10^5^ (∼20% confluence) cells stably expressing the μ-opioid receptors (MOR) (As described in the “before you begin” section) into various wells of a 12 well plate.
    ***Note:*** For every concentration of NAI to be tested, seed an additional well of cells to serve as a background control—which will be labeled with NAI-X in the presence of excess naloxone, 10 μM.
  b. Return cells to incubator set to 37°C, 93% humidity, and 5% CO_2_ for 48 h to grow to ∼1 × 10^6^ in number (70% confluence).
  c. Once cells have reached desired confluence, add a final concentration of 10 μM naloxone to all background control wells.
    **Caution:** NAI-X is less stable at basic (> pH 8); ensure media for labeling is pre-equilibrated at pH 7.4.
    **Caution:** Wells should contain equilibrated DMEM+10% FBS; there is no need to exchange for fresh media or remove FBS before labeling.
  d. Add a range of concentrations (10–500 nM) of NAI-X to control and test wells.
  e. Return cells to incubator at 37°C, 93% humidity, and 5% CO_2_. Allow labeling to proceed for 1 h.
  f. Once labeling is completed, wash each well once with PBS.
  g. Aspirate PBS and add 500 μL of TrypLE to lift cells. Incubate at room temperature for 4 min or 37°C for 2 min.
  h. Add 1 mL of Gibco™ High-Glucose DMEM with 10% FBS.
  i. Gently pipette up and down four times without introducing air bubbles to ensure all cells are lifted from the treated surface of the flask.
  j. Transfer cells to Eppendorf tubes.
  k. Spin down cells in Eppendorf tubes at 300 × *g* for 2 min.
  l. Remove supernatant and resuspend cells in 1 mL PBS with 10 μM naloxone.
    ***Note:*** Addition of naloxone in this step will remove the unreacted NAI-X from orthosteric binding site on the receptor and ensure the fluorescent signal is only from the NAI-X which has undergone covalent labeling.
  m. Analyze by flow cytometry, capturing 10,000 events after gating for single cells.
    ***Note:*** The results from above protocol will be used to construct a concentration response curve and calculate an EC_50_ concentration (see Figure 4A). This same protocol can be used to optimize the time of labeling, e.g., 400 nM of NAI-A488 (see Figure 4B) is used at various incubation times. A saturation curve is created to confirm specificity of labeling.

**Figure 3:**
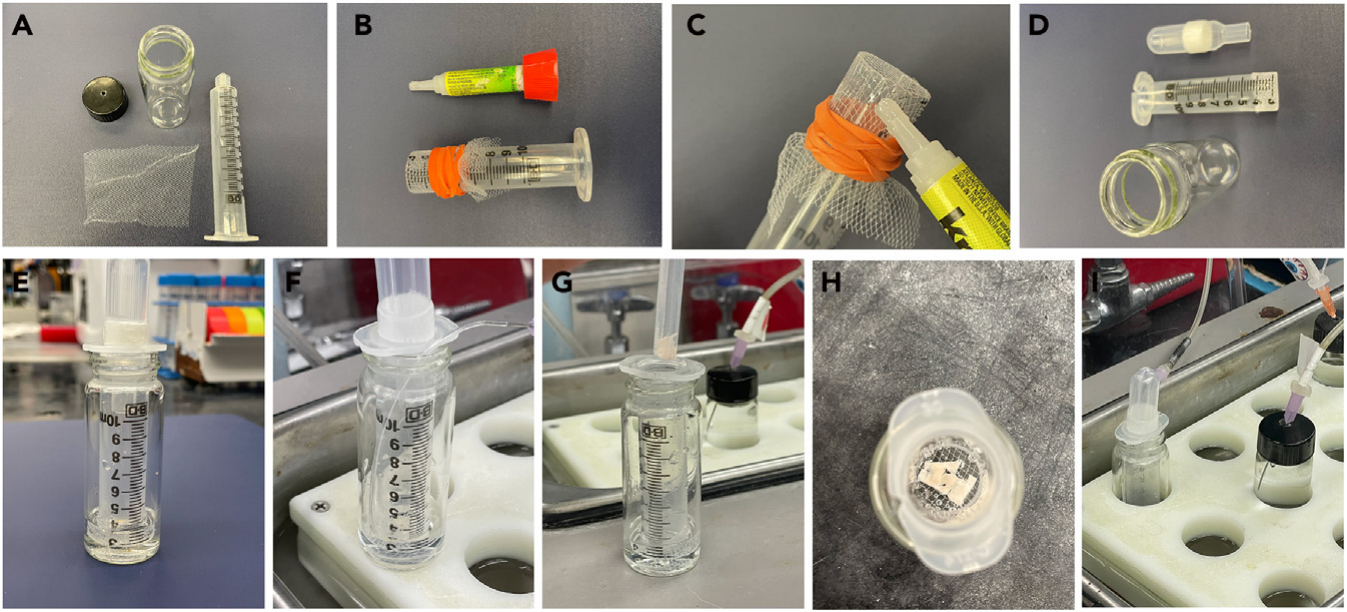
Incubation vial for brain slices (A–D) Materials needed to make a simple tool for holding brain slices at low ACSF volume e.g., 4 mL. (E–I) Setting up an incubation vial for labeling brain slices.

**Figure 4:**
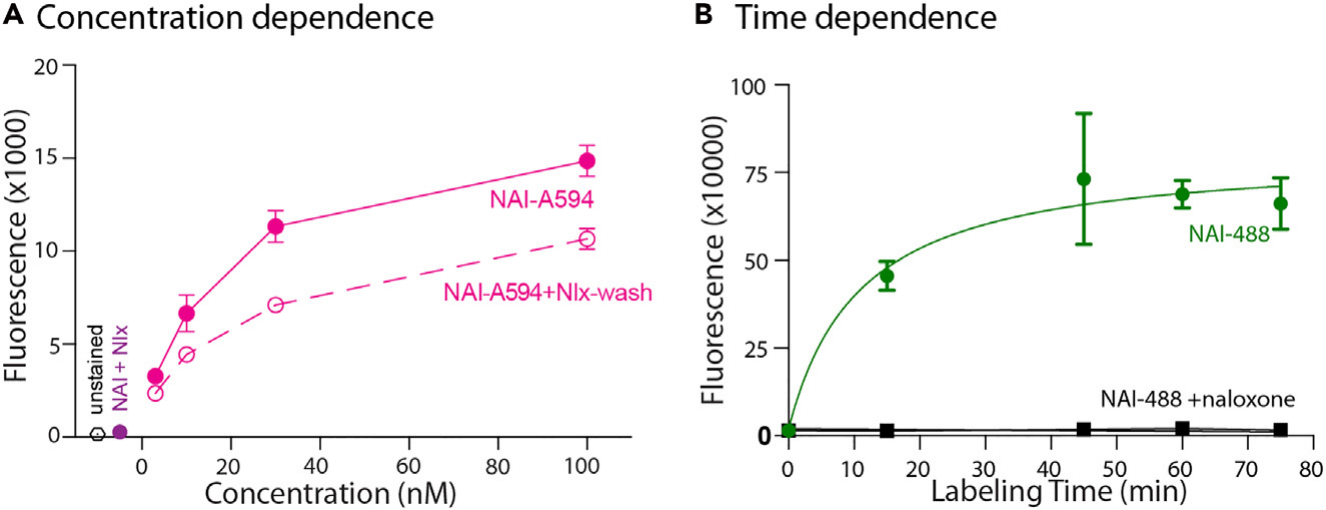
NAI labeling of HEK293 cells detected with flow cytometry (A) HEK293 cells expressed FMOR were incubated in NAI-A594 at various concentrations for 1 h. The labeling was completely blocked when 10 μM naloxone was included in the solution of 100 nM NAI-A594 (purple dot). Fluorescence increased with higher concentrations (solid red). The fluorescence was reduced when labeled cells were washed and left in 1 μM naloxone to displace the remaining NAI-A594 bound reversibly to the receptor.^1^ (B) Time dependence of labeling was monitored using cells incubated with 400 nM NAI-A488. The labeling was saturable in about 1 h (green line). Naloxone 10 μM was included in the solution of NAI-A488 at each time point to demonstrate a negligible background fluorescence due to non-specific binding (black line). Data from N = 3 independent biological replicates plotted with SD.

**Figure 5:**
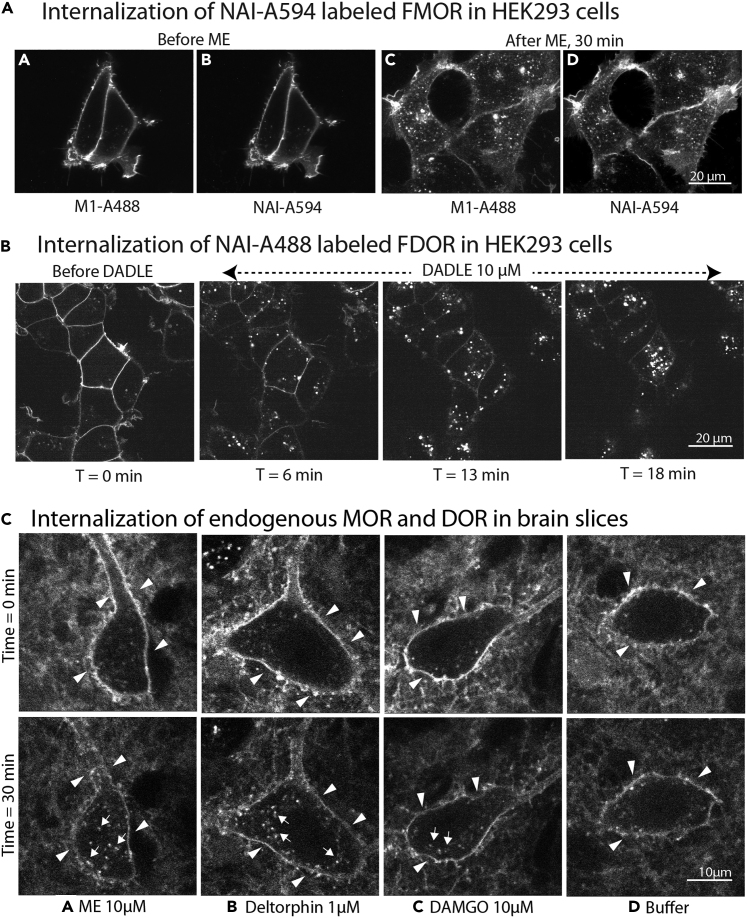
Agonist-induced receptor endocytosis (A) [Met^5^]enkephalin (ME) was used to activate and induce endocytosis of FMORs labeled with NAI-A594, which co-localized with anti-Flag (M1) conjugated to Alexa488.^1^ (B) Time-course of DADLE stimulated FDOR endocytosis, n = 5, representative example shown. (C) Agonist-dependent endocytosis of endogenous MOR and DOR in striatal cholinergic interneurons.^2^ Arrow heads indicate fluorescence staining along plasma membrane, which appears as a line. Arrows show some pools of endosomes as fluorescence puncta throughout the cytoplasm after agonist activation. A few fluorescent puncta were observed in cells before agonist application or after exposure with only ACSF. This phenomenon likely happened as the result of constitutive internalization.

7. Live cell imaging of Opioid Receptors stably overexpressed in cultured cells tagged with NAI-X.
  The following protocol describes the application of NAI-X in receptor internalization study.
  a. Plate cells on imaging dishes. We use LabTek II 8 well dishes with borosilicate glass bottoms.
    ***Note:*** Cells should be approximately 40%–60% confluent on day of imaging. We typically seed 3 × 10^4^ cells per well (∼10% confluence) on day one.
    **CRITICAL:** We have found that even though these LabTek II 8 well dishes are pre-coated, cells better adhere after additional coating with poly-L-lysine, following manufacturer’s instructions.
  b. Two days later, warm media to 37°C in incubator.
    ***Note:*** Optional, use imaging media without phenol red, and/or to supplement with HEPES if microscope does not include CO_2_ control. The pH of the medium should be around 7.4.
  c. Add NAI-A488 to well(s) at 400 nM.
  d. Return cells to incubator to label for 10 min.
  e. Wash cells twice with pre-equilibrated media (DMEM without phenol red, supplemented with 20 mM HEPES) and replace with fresh pre-equilibrated media.
  f. Image on microscope (Nikon Spinning Disk confocal with temperature control at 37°C, 100× objective, 2-s exposure per image).
  g. Add agonist and monitor on microscope for internalization. In Figure 5, images taken every 6 min over approximately 20 min.

### Validation of NAI-X labeling of opioid receptors in brain slices

**Timing: 2–4 h (for step 8)**

**Timing: 1 day (for step 9)**

NAI-X can fluorescently tag ORs in brain tissues of any animals. The labeling can be done *in situ* in acutely prepared slices. The labeled slices can be fixed for further study.

**Figure 6:**
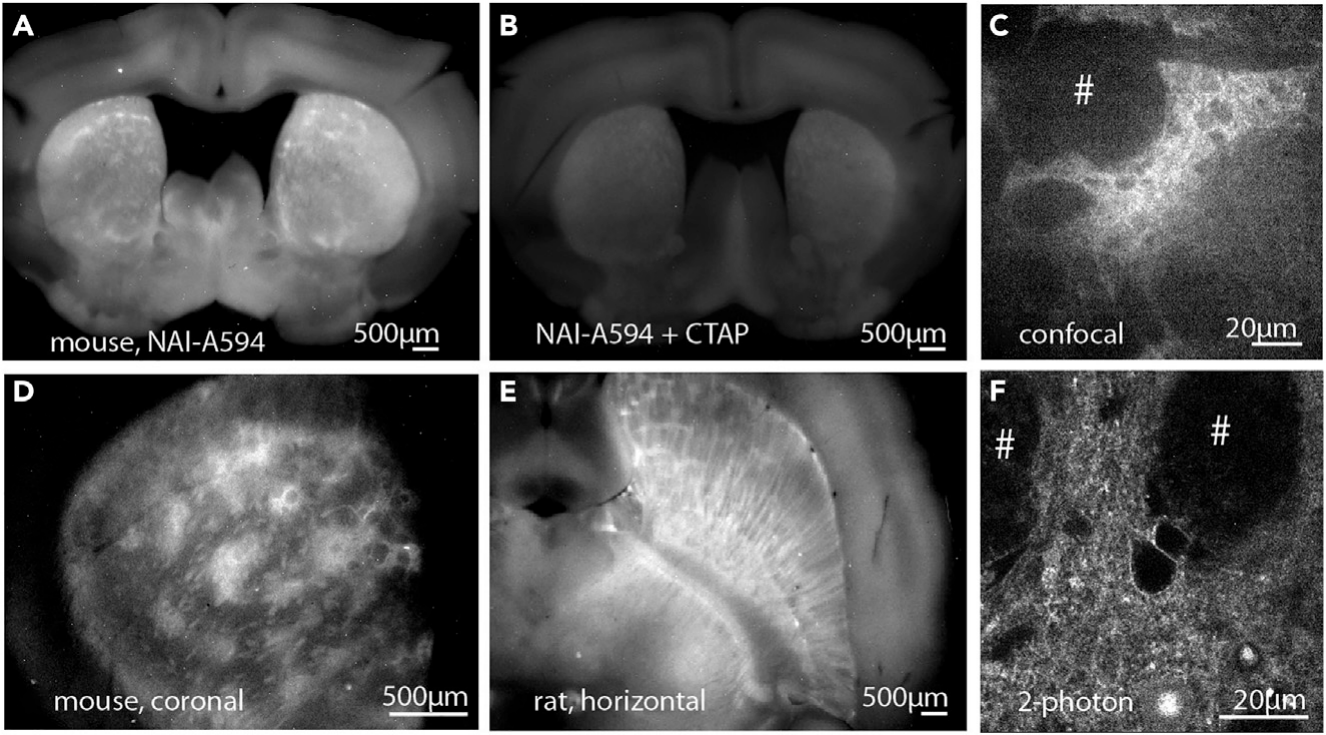
Visualization of labeled ORs in the acute (live) slices of striatum with different microscopes (A, B, D, and E) A macro zoom Olympus microscope was used to depict localization of labeled ORs in large areas (A, B, D, & E). Labeling of striatal patches was lacking when CTAP, a MOR selective antagonist was included (B). (C and F) Detailed staining along plasma membrane of neurons were photographed by a confocal or 2-photon microscope.^1^
  k. Take the labeled slice to a 2-photon microscope to visualize labeled receptors on a neuron.
  l. Submerge slice in continuous flow of oxygenated ACSF at 34°C.
    ***Note:*** A perfusion system provides a convenient way for drug application and washing steps.
  m. Take images using a 60× water immersion lens (Olympus LUMFI, NA 1.1) and laser at 810 nm excitation.

8. Labeling of opioid receptors in live brain slices.
  These steps describe the application of NAI-X in visualizing ORs in acute (freshly prepared) brain slices.
  a. Prepare brain slices as shown in the “before you begin” section.
    ***Note:*** We made a simple incubation vial (see Figure 3) for economical uses of NAI-X in labeling live brain slices. Most materials are available in the laboratory. The tool allows a small usage of NAI-X while maintaining healthy brain slices during labeling, and thus can be used in subsequent functional assays e.g., electrophysiological recording and receptor internalization.
  b. Make an incubating vial using a 10-mL syringe, a small piece of mesh fabric (2 × 2 inches) and a 30-mL scintillation vial (Figure 3A).
    i. Cut the tip of 10-mL syringe near the 3 mL mark.
    ii. Stretch the net cloth over the opening, secure the cloth with rubber band (Figure 3B).
    iii. Use cyanoacrylate glue (Krazy glue) to fix mesh fabric to barrel of syringe (Figure 3C).
    iv. Cut and remove the excess fabric material (Figure 3D).
    v. Handle of transfer pipet can be used as a convenient cap (Figures 3D and 3E).
  c. Add 4 mL ACSF buffer into the scintillation vial and set up the tool as shown in Figure 3E.
  d. Insert a small tube for bubbling carbogen gas (5% carbon dioxide + 95% oxygen).
    ***Note:*** The bubbling tube is outside net chamber, see Figure 3F.
  e. Add 4 μL of NAI-X to the vial. The concentration of NAI-X is 100 nM.
  f. Mix well.
  g. Drop a brain slice inside the net chamber, Figures 3G and 3H.
    **Caution:** More than one slice can be placed in the chamber, but they should not be stacked on top of each other.
  h. Incubate brain slices for 1 h at 30°C–34°C (Figure 3I).
    **Caution:** To avoid cell death during this step, be careful not to bubble too close and cause brain slices to swirl.
    ***Note:*** NAI-A594 is a good choice for two-photon microscopy because Alexa594 dye has a high two-photon absorption at 810 nm.
    ***Note:*** Similar to cell culture experiments, we recommend using naloxone to confirm specific labeling of ORs. When included in labeling solution of NAI-A594, there will be negligible fluorescent signals on brain slices. Naloxone can also be used to displace residual NAI-A594 in the binding pocket.
    **Caution:** If labeled neurons are subjected for further functional assays, it is important to ensure that naloxone used after labeling is completely washed out with ACSF buffer prior to experiments. Antagonist property of naloxone will hinder the action of test drugs.
  i. Remove a labeled slice and submerge it under ACSF in a petri dish.
  j. Take slices to a macroscope to visualize labeled receptors on large brain areas.
    ***Note:*** We used a Macro Zoom Olympus MVX10 microscope and a MV PLAPO2xC, NA 0.5 lens (Olympus) to visualize and capture images. A yellow LED (567 nm) is used for excitation of Alexa 594 dye. (See Figure 6 for examples).

9. Labeling of opioid receptors for fixed brain slices.
  These steps describe NAI-X labeling for fixed tissues.
  a. Prepare brain slices as shown in “before you begin” section.
  b. Incubate brain slices in oxygenated ACSF containing NAI-X at a final concentration of 30 or 100 nM for 1 h at 30°C–34°C.
  c. Wash the labeled slices, 2 × 10 mL in ACSF.
  d. Add 4% formaldehyde to labeled slices in Petri Dish.
    ***Note:*** The volume of 4% formaldehyde can be varied depending on the size of petri dish. Slices should be completely submerged under the fixative solution.
  e. Shake slices in a Petri dish using a rotator for 20 min.
  f. Wash fixed slices in a saline-phosphate buffer twice.
    ***Note:*** The volume of saline-phosphate can be varied depending on the size of petri-dish. Again slices should be completely submerged under buffer.
  g. Mount fixed slices using an antifade mounting medium (for examples: Prolong™, or Vectashield).
  h. Capture images using a confocal microscope. The example images (Figure 8) shown in this protocol were captured with a 20× air lens (Zeiss Plan Apochromic, NA 0.8) on a Zeiss LSM 980 with Airyscan 2 at 553 nm excitation and 568 nm emission.

**Figure 7:**
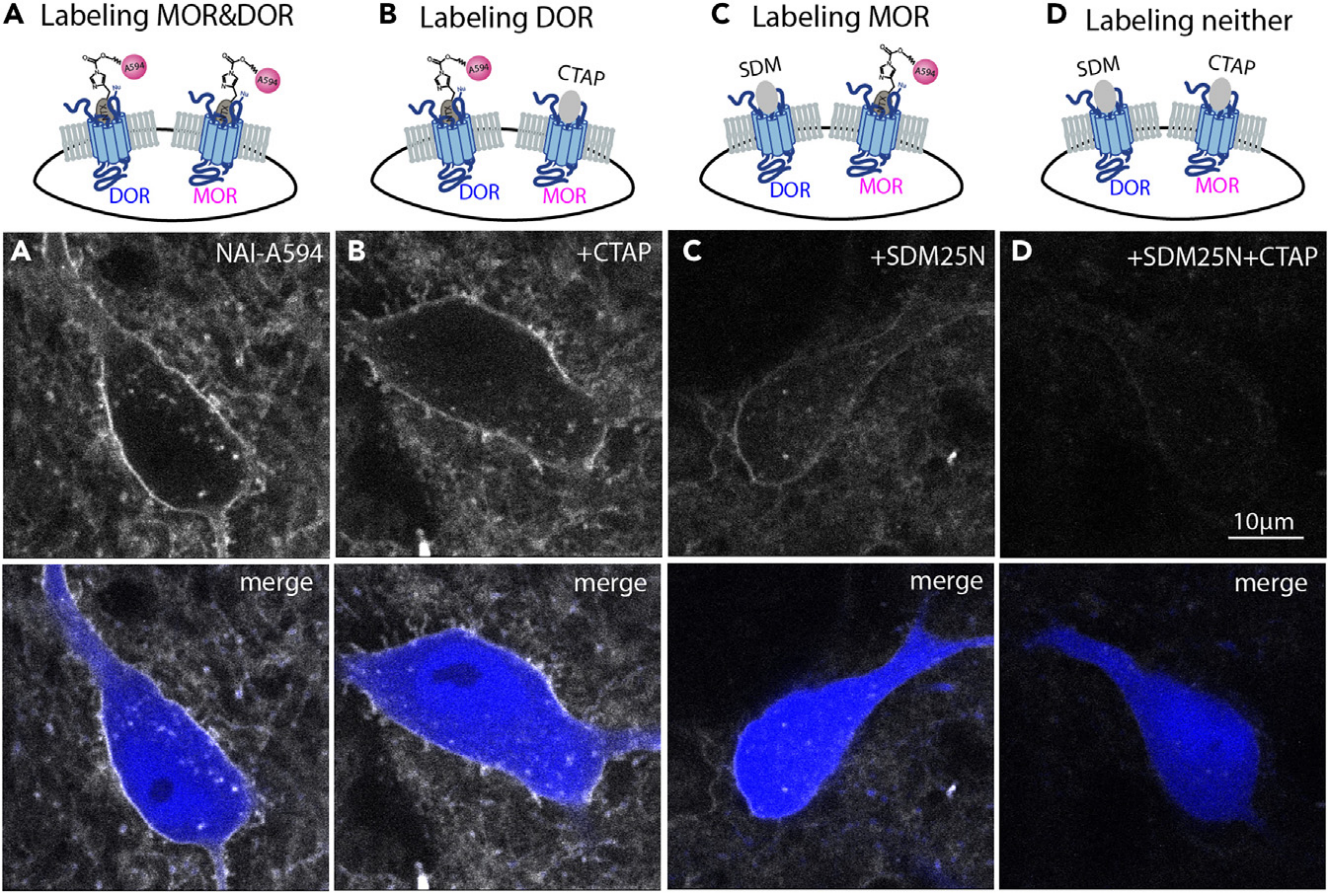
Blocking labeling of NAI-A594 with selective antagonists, CTAP and SDM25N showed the labeling of the other receptor subtype when both MOR and DOR are present on a single neuron of an acute (live) slice preparation ChAT(BAC)eGFP neurons in the striatum were colored in blue. (A) NAI-A594 labeled both MOR and DOR on a cholinergic interneuron.^2^ (B) Labeling of DOR was achieved with the use of CTAP, a MOR selective antagonist to block labeling of NAI-A594 at MOR.^2^ (C) Labeling of MOR with NAI-A594 was done by the blockade of DOR using SDM25N, a DOR selective antagonist.^2^ (D) No labeling of MOR and DOR with NAI-A594 when both CTAP and SDM25N were present.^2^

**Figure 8:**
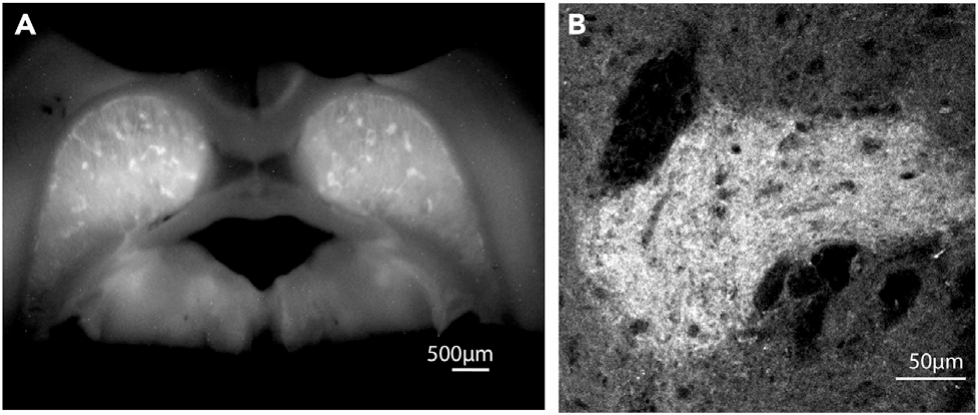
Visualization of labeled ORs in fixed slices (A and B) A slice was post-fixed in 4% PFA after labeling acute slices with NAI-A594. Images were taken from a macro zoom Olympus microscope (A) or a confocal microscope (B).

## Expected outcomes

NAI-X works to guide and covalently attach reporters like fluorescent dyes or biotin to ORs. The labeling property of NAI-X is concentration- and time-dependent indicating specificity to ORs. It is further confirmed by the blockade of labeling when co-incubating NAI-X in the presence of naloxone, a universal opioid antagonist (Figure 4). NAI-X has a relative high binding affinity of NAI-X to ORs hence it may linger in the orthosteric binding pocket of receptor for sometimes, especially after incubating in a saturating concentration of NAI-X. Adding naloxone to samples before flow cytometric measurement will ensure to eliminate the signal of this reversible binding NAI-X as well as verify the covalent tagging of fluorophores (Figure 4). Labeling of opioid receptors with NAI-X can be effectively achieved at EC_50_ concentrations of nanomolar ranges. We recommend using this low concentration, which can be easily washed with a buffer for labeling if the labeled receptors will be used later in functional assays.

Labeled ORs by NAI-X are still functional. Agonist-induced receptor endocytosis is a quick and reliable assay to check functionality of the labeled receptors. We observed MOR and DOR internalization in HEK293 cells expressing Flag-MOR and Flag-DOR, respectively using a confocal microscope (Figures 5A and 5B). With a 2-photon microscope, we observed internalization of endogenous DOR in mouse striatal cholinergic interneurons after application of [Met^5^]enkephalin (ME) and deltorphin. DAMGO, which is a MOR selective agonist did not induce observable MOR internalization.^2^

NAI-X is a useful tool for identifying neurons expressing ORs in live brain tissues.^1^ Visualization of ORs can be observed using various microscopes including a macroscope, a confocal microscope and a 2-photon microscope (Figure 6).

NAI-A594 can efficaciously label MOR and DOR. To differentiate if a neuron expresses each receptor or both, we used selective antagonist of each receptor e.g., CTAP for MOR and SDM25N for DOR to block labeling of each receptor subtype. Figure 7 shows examples of labeling OR subtypes on a single neuron co-expressing MOR and DOR of cholinergic interneurons from ChAT(BAC)-eGFP transgenic mice.^2^ The cholinergic interneurons from these mice expressed green fluorescent protein via the regulation of choline acetyltransferase gene (shown in Figure 7 as blue cells). Co-incubation of NAI-A594 with a selective MOR antagonist CTAP was used to display labeling of DOR on a cholinergic interneuron of the striatum. Alternatively, labeling MOR with NAI-A594 was done in the presence of a selective DOR antagonist SMD25N (Figure 7).

NAI-labeled ORs in brain slices can be fixable. This application is useful for anatomical studies. Figure 8 shows examples labeled ORs of fixed striatal slices. As labeling of this reagent relies on proper conformations of receptors, it is essential to label live brain tissues before fixation (see step 9).

## Limitations

Although NAI-X is a small molecule and can penetrate deeper in the tissues than antibodies, fluorescent signals significantly decrease after 100 μm from the top surface. The optimal working range for best signals is found to be between 10-50 μm from the surface.

## Troubleshooting

### Problem 1

Labeling receptors with NAI-X could potentially perturb receptor functions.

This is a common problem for any modified receptors including genetically or chemically tagged receptors that should be investigated. The linker in the NAI-X molecule was designed with the goal of allowing labeling of the receptor on its extracellular loops, thus minimizing the potential for the covalently bound fluorophore to disrupt receptor function that occurs via interaction with signaling proteins in the cytoplasm. We found the labeled receptors were functional as shown by activation of G protein-gated inwardly rectifying potassium channels of labeled MOR in a locus coeruleus neurons^1^, DOR internalization in cholinergic interneurons of the striatum^2^, and ME-induced MOR and DADLE-induced DOR internalization in HEK293 cells (Figures 5A and 5B). However, it remains possible that a fluorescent dye on the receptor may perturb the accessibility and interaction of agonists at the binding pocket. It is therefore important to systematically examine the functions of labeled receptors and compare the results with unlabeled receptors in each system of interest.

### Potential solution

Construct and compare EC_50_’s and maximal responses of labeled and unlabeled receptors in the assay of interest. The assays should include but not limit to recruitment of mini-G proteins and/or arrestins, activation of G protein-gated inwardly rectifying potassium channels, and production of cAMP. We recommend using a set of well-known full agonists and partial agonists to validate receptor functions.

### Problem 2

Trafficking of labeled receptors in the absence of agonist activation (see step 7).

A potential issue that could occur in visualization of NAI-X labeled ORs in cell culture is intracellular fluorescence signals before agonist activation of ORs. Importantly, NAI-A594 or NAI-A488 is not expected to permeabilize and label intracellular receptors. When long incubation of NAI compounds is done at 37°C, fluorescent puncta are observed without agonist activation. The problem is likely due to the labeled ORs undergoing constitutive endocytosis during incubation time.

### Potential solution

• Incubate cells with a high concentration of NAI-X for a shorter time e.g., 10 min. A near saturating concentration is recommended for this fast labeling. This is suitable for cell cultures where receptors on a monolayer of cells can quickly react with NAI-X. Additionally, the labeling with this high concentration of NAI-X can also be done at room temperature or on ice to prevent endocytosis. It is also important to keep in mind that NAI-X is a reversible antagonist and thus it is important to thoroughly wash this reagent from samples prior to functional assays.
• When brain slices are used, incubating slices near EC_50_ concentration of NAI-X is necessary. Brain slices contain various cell types, and a high concentration of NAI-X may cause substantial non-specific labeling. Incubating slices with NAI-X at room temperature can minimize constitutive endocytosis.

### Problem 3

Labeling fixed tissues (see step 9).

The labeling of NAI-X is dependent on proper conformations of the receptor for the ligand to bind and trigger the reaction. Labeling fixed cells or tissues with NAI-X will potentially yield a lower quality of image compared to labeling live tissues.

### Potential solution

Label freshly prepared brain slices before fixation process.

## Resource availability

### Lead contact

Further information and requests for resources and reagents should be directed to and will be fulfilled by the lead contact, Seksiri Arttamangkul (arttaman@ohsu.edu) and Braden Lobingier (lobingib@ohsu.edu).

### Materials availability

NAI-A594 and NAI-488 generated in this study can be made available on request to the Medicinal Chemistry Core Facility at Oregon Health and Science University. There will be fees for service depending on the amount of the material. Additionally, chemists at the core facility can customize the reagents for different reporters or fluorescent dyes. Please contact the core director, Dr. Aaron Nilsen (nilsena@ohsu.edu).

### Data and code availability

This study did not report datasets or original code.

## Supplemental information

Data S1. Chemical reactions of each synthesis step 1. **Reaction equation 1**, related to the synthesis of compound 1: 3-{[2-(1H-imidazol-4-yl)ethyl]carbamoyl}propanoic acid in step 1. 2. **Reaction equation 2**, related to the synthesis of compound 2: N-[(1S,5R,13R,14R,17S)-4-(cyclopropylmethyl)-10,17-dihydroxy-12-0xa-4-azapentacyclo[9.6.1.0^1,13^.0^5,17^.0^7,18^]octadeca-7,9,11(18)-trien-14-yl]-N’-[2-(1H-imidazol-4-yl)ethylbutanediamid in step 2. 3. **Reaction equation 3**, related to the synthesis of compound 3: 2,5-dioxopyrrolidin-1-yl 2-(pro-2-yn-1-yloxy)ethyl carbonate in step 3. 4. **Reaction equation 4**, related to the synthesis of compound 4: 2-(prop-2-yn-1-yloxy)ethyl 4-[2-(3-{[1s,5R,13R,14R)-4-(cyclopropylmethyl)-10,17-dihydroxy-12-oxa-4-azapentacyclo[9.6.1.0^1,13^.0^5,17^.0^7,18^] octadeca-7,9,11(18)-trien-14-yl]carbamoyl}propanamido)ethyl]-1H-imidazol-1-carboxylate in step 4. 5. **Reaction equation 5**, related to the conjugation of NAI-AK with Alexa594 azide using copper (I)-catalyzed azide-alkyne cycloaddition (CuAAA) click chemistry in step 5.
