# Supplementary figures and images for: Ligand-directed labeling of opioid receptors for covalent attachment of fluorophores or small-molecule probes
Source: STAR Protoc. 2023 Apr 26;4(2):102231. doi: 10.1016/j.xpro.2023.102231 (PMC10154970; doi:10.1016/j.xpro.2023.102231)

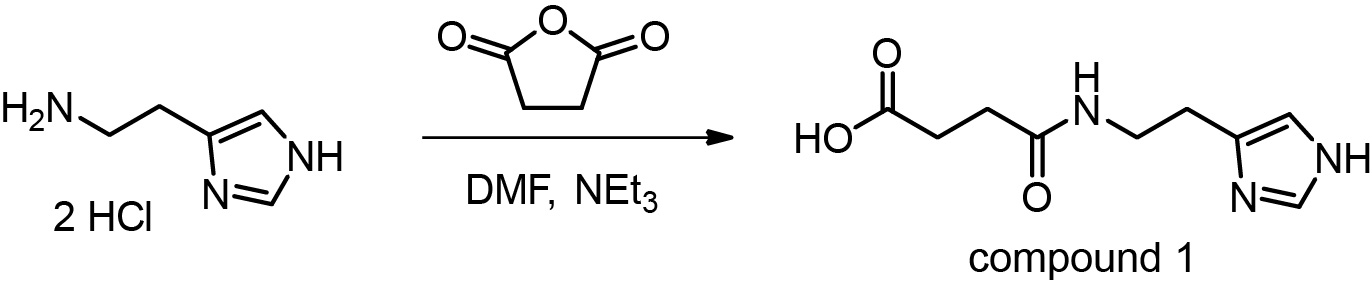

Supplement: Data S1. Chemical reactions of each synthesis step — 1. Reaction equation 1, related to the synthesis of compound 1: 3-{[2-(1H-imidazol-4-yl)ethyl]carbamoyl}propanoic acid in step 1. 2. Reaction equation 2, related to the synthesis of compound 2: N-[(1S,5R,13R,14R,17S)-4-(cyclopropylmethyl)-10,17-dihydroxy-12-0xa-4-azapentacyclo[9.6.1.01,13.05,17.07,18]octadeca-7,9,11(18)-trien-14-yl]-N’-[2-(1H-imidazol-4-yl)ethylbutanediamid in step 2. 3. Reaction equation 3, related to the synthesis of compound 3: 2,5-dioxopyrrolidin-1-yl 2-(pro-2-yn-1-yloxy)ethyl carbonate in step 3. 4. Reaction equation 4, related to the synthesis of compound 4: 2-(prop-2-yn-1-yloxy)ethyl 4-[2-(3-{[1s,5R,13R,14R)-4-(cyclopropylmethyl)-10,17-dihydroxy-12-oxa-4-azapentacyclo[9.6.1.01,13.05,17.07,18] octadeca-7,9,11(18)-trien-14-yl]carbamoyl}propanamido)ethyl]-1H-imidazol-1-carboxylate in step 4. 5. Reaction equation 5, related to the conjugation of NAI-AK with Alexa594 azide using copper (I)-catalyzed azide-alkyne cycloaddition (CuAAA) click chemistry in step 5. [file mmc1.zip › Reaction equation 1.jpg]

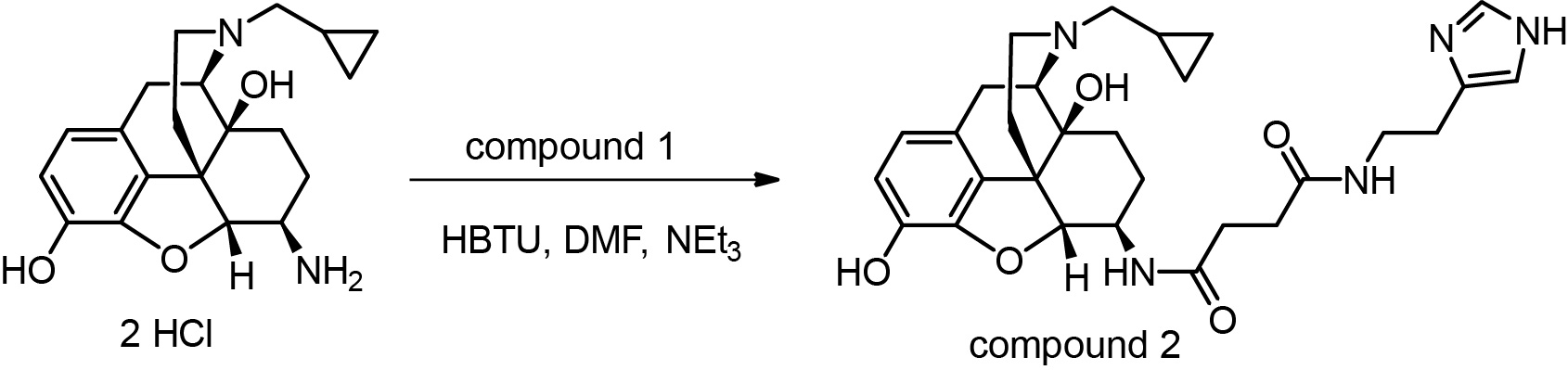

Supplement: Data S1. Chemical reactions of each synthesis step — 1. Reaction equation 1, related to the synthesis of compound 1: 3-{[2-(1H-imidazol-4-yl)ethyl]carbamoyl}propanoic acid in step 1. 2. Reaction equation 2, related to the synthesis of compound 2: N-[(1S,5R,13R,14R,17S)-4-(cyclopropylmethyl)-10,17-dihydroxy-12-0xa-4-azapentacyclo[9.6.1.01,13.05,17.07,18]octadeca-7,9,11(18)-trien-14-yl]-N’-[2-(1H-imidazol-4-yl)ethylbutanediamid in step 2. 3. Reaction equation 3, related to the synthesis of compound 3: 2,5-dioxopyrrolidin-1-yl 2-(pro-2-yn-1-yloxy)ethyl carbonate in step 3. 4. Reaction equation 4, related to the synthesis of compound 4: 2-(prop-2-yn-1-yloxy)ethyl 4-[2-(3-{[1s,5R,13R,14R)-4-(cyclopropylmethyl)-10,17-dihydroxy-12-oxa-4-azapentacyclo[9.6.1.01,13.05,17.07,18] octadeca-7,9,11(18)-trien-14-yl]carbamoyl}propanamido)ethyl]-1H-imidazol-1-carboxylate in step 4. 5. Reaction equation 5, related to the conjugation of NAI-AK with Alexa594 azide using copper (I)-catalyzed azide-alkyne cycloaddition (CuAAA) click chemistry in step 5. [file mmc1.zip › Reaction equation 2.jpg]

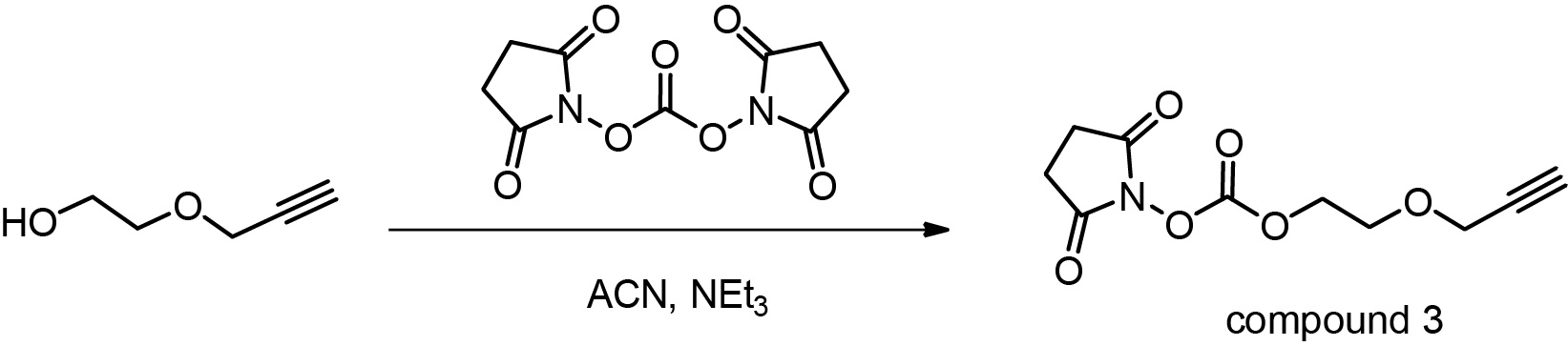

Supplement: Data S1. Chemical reactions of each synthesis step — 1. Reaction equation 1, related to the synthesis of compound 1: 3-{[2-(1H-imidazol-4-yl)ethyl]carbamoyl}propanoic acid in step 1. 2. Reaction equation 2, related to the synthesis of compound 2: N-[(1S,5R,13R,14R,17S)-4-(cyclopropylmethyl)-10,17-dihydroxy-12-0xa-4-azapentacyclo[9.6.1.01,13.05,17.07,18]octadeca-7,9,11(18)-trien-14-yl]-N’-[2-(1H-imidazol-4-yl)ethylbutanediamid in step 2. 3. Reaction equation 3, related to the synthesis of compound 3: 2,5-dioxopyrrolidin-1-yl 2-(pro-2-yn-1-yloxy)ethyl carbonate in step 3. 4. Reaction equation 4, related to the synthesis of compound 4: 2-(prop-2-yn-1-yloxy)ethyl 4-[2-(3-{[1s,5R,13R,14R)-4-(cyclopropylmethyl)-10,17-dihydroxy-12-oxa-4-azapentacyclo[9.6.1.01,13.05,17.07,18] octadeca-7,9,11(18)-trien-14-yl]carbamoyl}propanamido)ethyl]-1H-imidazol-1-carboxylate in step 4. 5. Reaction equation 5, related to the conjugation of NAI-AK with Alexa594 azide using copper (I)-catalyzed azide-alkyne cycloaddition (CuAAA) click chemistry in step 5. [file mmc1.zip › Reaction equation 3.jpg]

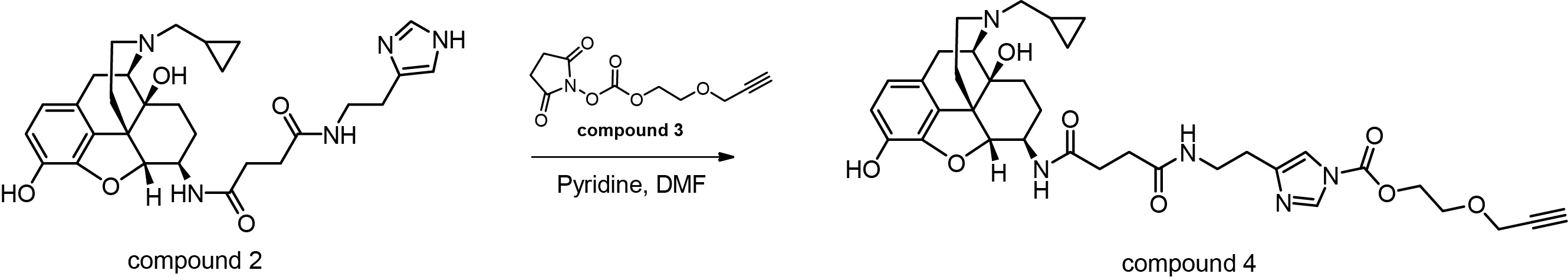

Supplement: Data S1. Chemical reactions of each synthesis step — 1. Reaction equation 1, related to the synthesis of compound 1: 3-{[2-(1H-imidazol-4-yl)ethyl]carbamoyl}propanoic acid in step 1. 2. Reaction equation 2, related to the synthesis of compound 2: N-[(1S,5R,13R,14R,17S)-4-(cyclopropylmethyl)-10,17-dihydroxy-12-0xa-4-azapentacyclo[9.6.1.01,13.05,17.07,18]octadeca-7,9,11(18)-trien-14-yl]-N’-[2-(1H-imidazol-4-yl)ethylbutanediamid in step 2. 3. Reaction equation 3, related to the synthesis of compound 3: 2,5-dioxopyrrolidin-1-yl 2-(pro-2-yn-1-yloxy)ethyl carbonate in step 3. 4. Reaction equation 4, related to the synthesis of compound 4: 2-(prop-2-yn-1-yloxy)ethyl 4-[2-(3-{[1s,5R,13R,14R)-4-(cyclopropylmethyl)-10,17-dihydroxy-12-oxa-4-azapentacyclo[9.6.1.01,13.05,17.07,18] octadeca-7,9,11(18)-trien-14-yl]carbamoyl}propanamido)ethyl]-1H-imidazol-1-carboxylate in step 4. 5. Reaction equation 5, related to the conjugation of NAI-AK with Alexa594 azide using copper (I)-catalyzed azide-alkyne cycloaddition (CuAAA) click chemistry in step 5. [file mmc1.zip › Reaction equation 4.jpg]

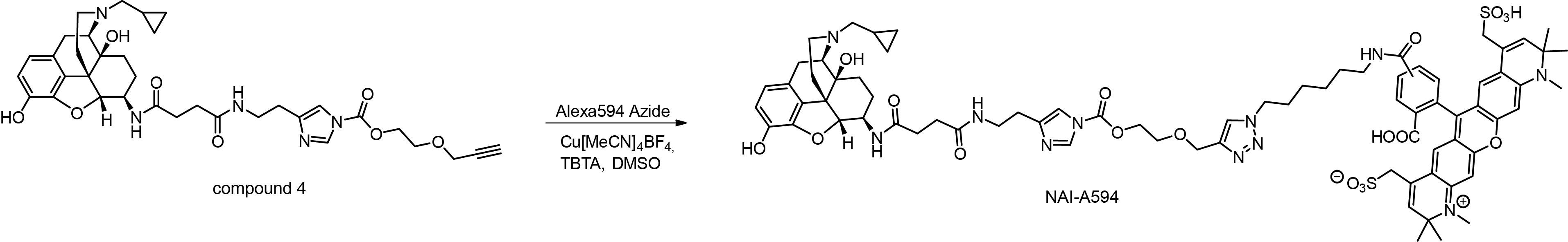

Supplement: Data S1. Chemical reactions of each synthesis step — 1. Reaction equation 1, related to the synthesis of compound 1: 3-{[2-(1H-imidazol-4-yl)ethyl]carbamoyl}propanoic acid in step 1. 2. Reaction equation 2, related to the synthesis of compound 2: N-[(1S,5R,13R,14R,17S)-4-(cyclopropylmethyl)-10,17-dihydroxy-12-0xa-4-azapentacyclo[9.6.1.01,13.05,17.07,18]octadeca-7,9,11(18)-trien-14-yl]-N’-[2-(1H-imidazol-4-yl)ethylbutanediamid in step 2. 3. Reaction equation 3, related to the synthesis of compound 3: 2,5-dioxopyrrolidin-1-yl 2-(pro-2-yn-1-yloxy)ethyl carbonate in step 3. 4. Reaction equation 4, related to the synthesis of compound 4: 2-(prop-2-yn-1-yloxy)ethyl 4-[2-(3-{[1s,5R,13R,14R)-4-(cyclopropylmethyl)-10,17-dihydroxy-12-oxa-4-azapentacyclo[9.6.1.01,13.05,17.07,18] octadeca-7,9,11(18)-trien-14-yl]carbamoyl}propanamido)ethyl]-1H-imidazol-1-carboxylate in step 4. 5. Reaction equation 5, related to the conjugation of NAI-AK with Alexa594 azide using copper (I)-catalyzed azide-alkyne cycloaddition (CuAAA) click chemistry in step 5. [file mmc1.zip › Reaction equation 5.jpg]
